# How to Assess Risk Factors for Lead Dislodgement in Patients Receiving Cardiac Implantable Electronic Devices

**DOI:** 10.1002/clc.70131

**Published:** 2025-04-14

**Authors:** Naoya Kataoka, Teruhiko Imamura

**Affiliations:** ^1^ Second Department of Internal Medicine University of Toyama Toyama Japan

## Disclosure

The authors have nothing to report.

To the Editor,

Based on the hypothesis that focal inflammation following cardiac implantable electronic device (CIED) implantation is pivotal in the development of adhesions between the lead and surrounding tissues, Matsuda et al. demonstrated a higher incidence of lead dislodgement in patients undergoing immunosuppressive therapy [1]. Several concerns have been raised regarding this finding.

The causality between immunosuppressive therapy and lead dislodgement remains questionable. The tip of a CIED lead typically elutes steroids to prevent an increase in the pacing threshold immediately postimplantation, thereby mitigating acute device‐related inflammation. Consequently, focal inflammation is suppressed regardless of systemic immunosuppressant administration. Furthermore, many patients with cardiac sarcoidosis, who often require CIEDs and receive steroid therapy, do not exhibit a notably high incidence of lead dislodgement.

Alternative factors, such as frailty, might be implicated in lead dislodgement rather than steroid administration. Previous literature has identified frailty as an independent risk factor for lead dislodgement, potentially due to psychomotor agitation, inappropriate limb and chest movements, traumatic events, and progressive weight loss [2]. Long‐term steroid therapy is generally associated with the progression of frailty [3]. Did the authors evaluate the severity of frailty in individuals receiving steroid therapy?

In a representative case of lead dislodgement presented in the authors' study, the lead appears to be pulled upwards, indicating insufficient pre‐deflection at the time of implantation [1]. Variations in operator learning curves may have influenced clinical outcomes, including the incidence of lead dislodgement.

## Data Availability

The authors have nothing to report.

## References

[1] Y. Matsuda , M. Masuda , M. Asai , et al., “Impact of Immunosuppressive Therapy on Lead Dislodgement After Cardiac Implantable Electronic Device Implantation,” Clinical Cardiology 47, no. 6 (2024): e24310. 38888132 10.1002/clc.24310 PMC11184469

[2] R. Mlynarski , A. Mlynarska , M. Joniec , et al., “Predictors of Early Cardiac Implantable Electronic Device Lead Dislodgement in the Elderly,” International Journal of Environmental Research and Public Health 19, no. 22 (2022): 14766. 36429483 10.3390/ijerph192214766 PMC9690924

[3] K. Ryu , Y. Fukutomi , E. Nakatani , et al., “Frailty and Muscle Weakness in Elderly Patients With Asthma and Their Association With Cumulative Lifetime Oral Corticosteroid Exposure,” Allergology International 72, no. 2 (2023): 252–261. 36371246 10.1016/j.alit.2022.10.005

